# Local ferroelectric polarization switching driven by nanoscale distortions in thermoelectric ${\text {Sn}}_{0.7}{\text {Ge}}_{0.3}{\text {Te}}$

**DOI:** 10.1038/s41598-021-96299-3

**Published:** 2021-08-25

**Authors:** Aastha Vasdev, Moinak Dutta, Shivam Mishra, Veerpal Kaur, Harleen Kaur, Kanishka Biswas, Goutam Sheet

**Affiliations:** 1 Department of Physical Sciences, Indian Institute of Science Education and Research Mohali, Sector 81, S. A. S. Nagar, Manauli, PO 140306, Mohali, Punjab India; 2 New Chemistry Unit, Jawaharlal Nehru Centre for Advanced Scientific Research, Bengaluru, Karnataka India

**Keywords:** Physics, Condensed-matter physics, Ferroelectrics and multiferroics

## Abstract

A remarkable decrease in the lattice thermal conductivity and enhancement of thermoelectric figure of merit were recently observed in rock-salt cubic SnTe, when doped with germanium (Ge). Primarily, based on theoretical analysis, the decrease in lattice thermal conductivity was attributed to local ferroelectric fluctuations induced softening of the optical phonons which may strongly scatter the heat carrying acoustic phonons. Although the previous structural analysis indicated that the local ferroelectric transition temperature would be near room temperature in ${\text {Sn}}_{0.7}{\text {Ge}}_{0.3}{\text {Te}}$, a direct evidence of local ferroelectricity remained elusive. Here we report a direct evidence of local nanoscale ferroelectric domains and their switching in ${\text {Sn}}_{0.7}{\text {Ge}}_{0.3}{\text {Te}}$ using piezoeresponse force microscopy(PFM) and switching spectroscopy over a range of temperatures near the room temperature. From temperature dependent (250–300 K) synchrotron X-ray pair distribution function (PDF) analysis, we show the presence of local off-centering distortion of Ge along the rhombohedral direction in global cubic ${\text {Sn}}_{0.7}{\text {Ge}}_{0.3}{\text {Te}}$. The length scale of the ${\text {Ge}}^{2+}$ off-centering is 0.25–0.10 Å near the room temperatures (250–300 K). This local emphatic behaviour of cation is the cause for the observed local ferroelectric instability, thereby low lattice thermal conductivity in ${\text {Sn}}_{0.7}{\text {Ge}}_{0.3}{\text {Te}}$.

## Introduction

It is known that almost two-thirds of all energy-intensive processes release heat as a waste product. Therefore, the ability to convert heat into an useful form of energy is crucial. Thermoelectric materials are able to convert waste heat into electricity and thus find applicability in future energy management strategies^1–4^. The efficiency of heat to electricity conversion is given by the thermoelectric figure of merit (*zT*) which is:

$$zT=\frac{S^2\sigma }{\kappa } {\text T}$$

where, S, $\sigma$ and $\kappa$ denotes Seebeck co-efficient of a material, electrical conductivity and thermal conductivity respectively. The $\kappa$ in crystalline solids is mainly propagated via charge carriers ($\kappa _{el}$) and lattice vibrations ($\kappa _{lat}$). An ideal thermoelectric material should have high power factor (${\text {S}}^2\sigma$) and low $\kappa _{lat}$ and in this regard several routes have been found to be successful which can optimise these parameters^5^. Strategies like band convergence^6^, resonance level^7–9^, charge carrier optimization^10^ has been found to be effective to improve the power factor of a material while point defect scattering^11^, nano-structuring^12^, all-scale hierarchical architectures^13^ and compounds with intrinsically low $\kappa _{lat}$^14^ are found to be effective to lower the phonon transport. All these strategies led to development of some of the highest performing thermoelectric materials in the last decade^15–17^.

SnTe has been on the forefront in the thermoelectric research for the last decade due to its safer alternative to the toxic PbTe compounds, mainly due to its similar crystal and electronic structure^18^. Several SnTe based high performance thermoelectric materials has been developed primarily from the aforementioned strategies^6,19–23^. However, the major disadvantage has been the simultaneous optimization of both of these phonon properties. Recently, it has been observed that engineering ferroelectric instability in SnTe reduces its lattice (phonon) thermal conductivity enormously (0.67 W/mK at 300 K) while simultaneously retaining its high carrier mobility through the dielectric charge carrier screening^19^. SnTe has a non-centrosymmetric rhombohedral (R3m) phase below 100 K which transforms to global rock-salt cubic phase (Fm-3m) above 100 K^9,24,25^. Therefore, global ferroelectric ordering is not expected above 100 K in SnTe. Later, weak local ferroelectric instability was reported in SnTe near room temperature, which was attributed to condensation of triply degenerate unstable optical phonon modes in the cubic rock-salt phase^26^. 30 mol% Ge alloying in SnTe provides strong ferroelectric instability near room temperature (${\text {T}}_c \sim$ 290 K), which is accompanied via local chain-like off-centering of ${\text {Ge}}^{2+}$ in the global cubic SnTe^19^. This ferroelectric instability softens the polar transverse optical (TO) phonons, which couples with the heat carrying acoustic phonons, and in the process reduces the acoustic phonon lifetime^19^.

Previous structural studies have shown that Sn remains in a distorted octahedra inside the globally rocksalt cubic structure at high temperatures beyond its ferroelectric transition temperature^25,27^. This local off-centering of Sn is found to increase with increase in temperature, a rare occurrence termed as “emphanisis”^25^. GeTe in its ferroelectric phase is also found to be locally distorted along the [111] direction by a larger magnitude of $\sim 0.30$ Å and the local distortion remains unchanged beyond its ferroelectric transition temperature ($\sim 705$ K)^25,28^. Thus, it is imperative to investigate the structural identity of the 30 mol% GeTe–SnTe alloy before and beyond the ferroelectric instability temperature.

The presence of such local off-centering has benefited the thermoelectric performance of many compounds by lowering their $\kappa _{lat}$^29–32^. In ${\text {Sn}}_{0.7}{\text {Ge}}_{0.3}{\text {Te}}$ particularly, the local off-centering of cations are found to exist till the measured temperature of $\sim 2{\text {T}}_c$ (600 K). This explains the flat $\kappa _{lat}$ of ${\text {Sn}}_{0.7}{\text {Ge}}_{0.3}{\text {Te}}$ throughout the measured temperature^19^. However, the direct evidence of this local off-centering and its effect on the ferroelectric instability near the transition temperature has been lacking and this work unambiguously proves it.

## Results and discussion

Here, using temperature dependent piezoresponse force microscopy, we have observed local polarization switching in ${\text {Sn}}_{0.7}{\text {Ge}}_{0.3}{\text {Te}}$ near the room temperatures, indicating local ferroelectric instability—although global ferroelectric ordering has not been observed. We also observe clear butterfly loops in local strain vs applied electric field which is a tell-tale signature of piezoelectricity. The absence of global ferroelectric ordering indicates that the local ferroelectric switching might arise from local distortion in ${\text {Sn}}_{0.7}{\text {Ge}}_{0.3}{\text {Te}}$, rather than a global structural change. To investigate the local structural evolution of ${\text {Sn}}_{0.7}{\text {Ge}}_{0.3}{\text {Te}}$, we have also performed temperature dependent (250–300 K) synchrotron X-ray Pair Distribution Function (PDF) analysis. PDF analysis yielded a local off-centering of cations throughout the measured temperature range although the global structure of SnTe remains rock-salt. Local off-centering of Ge in the rhombohedral direction of SnTe gives rise to ferroelectric instability, thereby resulting in ultra-low lattice thermal conductivity and high thermoelectric performance.

In our PFM setup shown in Fig. S1a, we have a conductive cantilever in contact with the sample. A laser is directed on the cantilever and is reflected back to a quadrupole photo diode. A sinusoidal AC voltage ${\text {V}}_{ac}$ is applied to a piezo-chip attached to the cantilever to drive the cantilever into oscillation.The cantilever is then tuned at the contact-mode resonance frequency. All the measurements are then carried out at this resonance frequency in order to achieve maximum sensitivity^33–35^. In contact mode, the deflection of the cantilever is tracked while scanning it over the surface. PFM measures the mechanical response to the electric field applied between the tip and sample. The sample deforms i.e. locally expands or contracts in response to applied voltage, which in turn changes the deflection of cantilever. This deflection is interpreted in terms of a piezo-response signal. For PFM spectroscopy, a combination of AC and DC voltage of the form ${\text {V}}_{tip}$ = ${\text {V}}_{dc}$ + ${\text {V}}_{ac}$ sin($\omega$t) is applied to the tip. A piezoelectric sample deforms below the tip in presence of applied voltage. The response signal is interpreted in terms of phase, the first harmonic component of the tip deflection. If the polarization direction below the tip is in the same direction as the applied DC voltage, the sample would locally expand and vice versa. So, a sweeping DC voltage would show a switching behaviour in phase. This can be observed as hysteric phase switching in $\phi$ vs ${\text {V}}_{dc}$ curve. The amplitude of deformation can also be interpreted in the form A  = ${\text {A}}_0$ + ${\text {A}}_\omega$ cos($\omega$t + $\phi$). This is observed as butterfly loop in ${\text {A}}_\omega$ vs ${\text {V}}_{dc}$ curve. It should be noted that the hysteretic behaviour can be observed for reasons other than piezoelectricity such as electrostatic and electrochemical effects. In order to mitigate the electrostatic effects, we supply a sequence of DC voltages with AC modulation in triangular saw tooth pattern^36,37^. This switching spectroscopy protocol allows us to measure the response signal in “off” state of pulses. The growth of nanoscale topographic features, if any, indicate electrochemical reaction over the surface^35^.

For PFM spectroscopic measurements, we took a 4 mm $\times$ 4 mm $\times$ 1 mm ${\text {Sn}}_{0.7}{\text {Ge}}_{0.3}{\text {Te}}$ sample and placed it over a metallic plate connected to a heating Peltier cooling and heating stage. This stage, in turn, is connected to a common ground of a high voltage amplifier. The temperature of the metallic plate can be varied from 278 to 400 K. A conductive AFM tip, made of silicon coated with platinum, was brought in contact with the sample. The free air resonant frequency was 73 kHz. The resonant frequency in contact with the sample was found to vary between 260 and 300 kHz. The spring constant of the cantilever was found to be 2.1 N/m.

**Figure 1:**
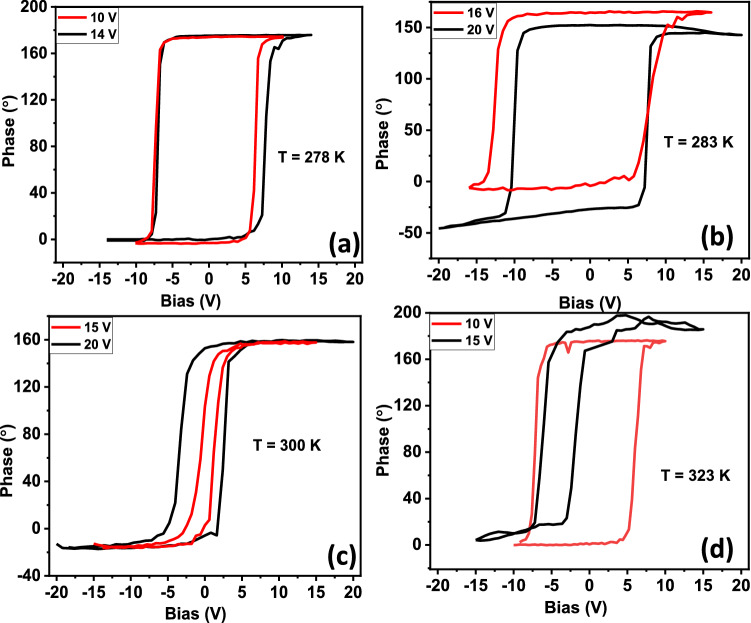
Phase vs ${\text {V}}_{dc}$ switching curve at temperature (**a**) 278 K (**b**) 283 K (**c**) 300 K and (**d**) 323 K.

In Fig. 1, we show the temperature dependence of phase vs ${\text {V}}_{dc}$ curve. The corresponding butterfly loop like amplitude vs field curve is shown in Fig. S2. The hysteretic switching by $180^{\circ }$ is clearly seen in Fig. 1a. At this temperature, the switching behaviour is very robust and does not depend upon the maximum voltage applied during the measurement. We clarify this by showing two representative curves captured with two maximum applied voltages. For the red curve, the maximum applied voltage was 10 V and for the black curve, the maximum applied voltage was 14 V. Similarly, as shown in Fig. 1b, at 283 K the difference of the respective hysteresis loops obtained by applying two different voltages (15 V and 20 V), was not significant in terms of the coercive fields, despite their slightly larger difference in overall shape. These two measurements were done at the temperatures lower than the predicted ferroelectric transition temperature ($\sim 290$ K). At 300 K , which is above the estimated transition temperature, the hysteresis loops appear only at certain points on the sample surface. But, they appear with a much smaller coercive voltage (Fig. 1c). Furthermore, at this temperature, the shape of the loop and the coercive field change dramatically when the maximum applied voltage is varied during the measurement. This deviation is even clearer in Fig. 1d where we present the data at 323 K, far above the estimated transition temperature. These observations indicate that while the hysteresis loops appear over a large temperature range, they are less dependent on the maximum applied voltages at lower temperatures. This dependence is more prominent at temperatures above 290 K, the estimated transition temperature. This help us conclude that the hysteresis loops appear dominantly from ferroelectricity at lower temperatures (T = 278 K and T = 283 K). This is consistent with the fact that the ferroelectric response is insensitive to the maximum voltage applied as long as the applied voltage is greater than the saturation voltage.

It should be noted that at higher temperatures, spurious factors like local electrochemical reaction might give rise to hysteresis loops which is expected to depend strongly on the maximum applied voltage. To re-emphasize on this point, as it is known, hysteretic polarization switching might arise due to electrochemical reaction between the tip and sample. To verify this, the area under the tip is scanned before and after the spectroscopic measurements. Growth of nanostructure would indicate local electrochemical reaction. Such nanostructure growth were not visible in topographic images after spectroscopic measurements at lower temperatures. However, at higher temperatures, signature of local ferroelectric instability arises probably due to the local cation (${\text {Ge}}^{2+}$) off-centering in global cubic rock-salt SnTe lattice.

**Figure 2:**
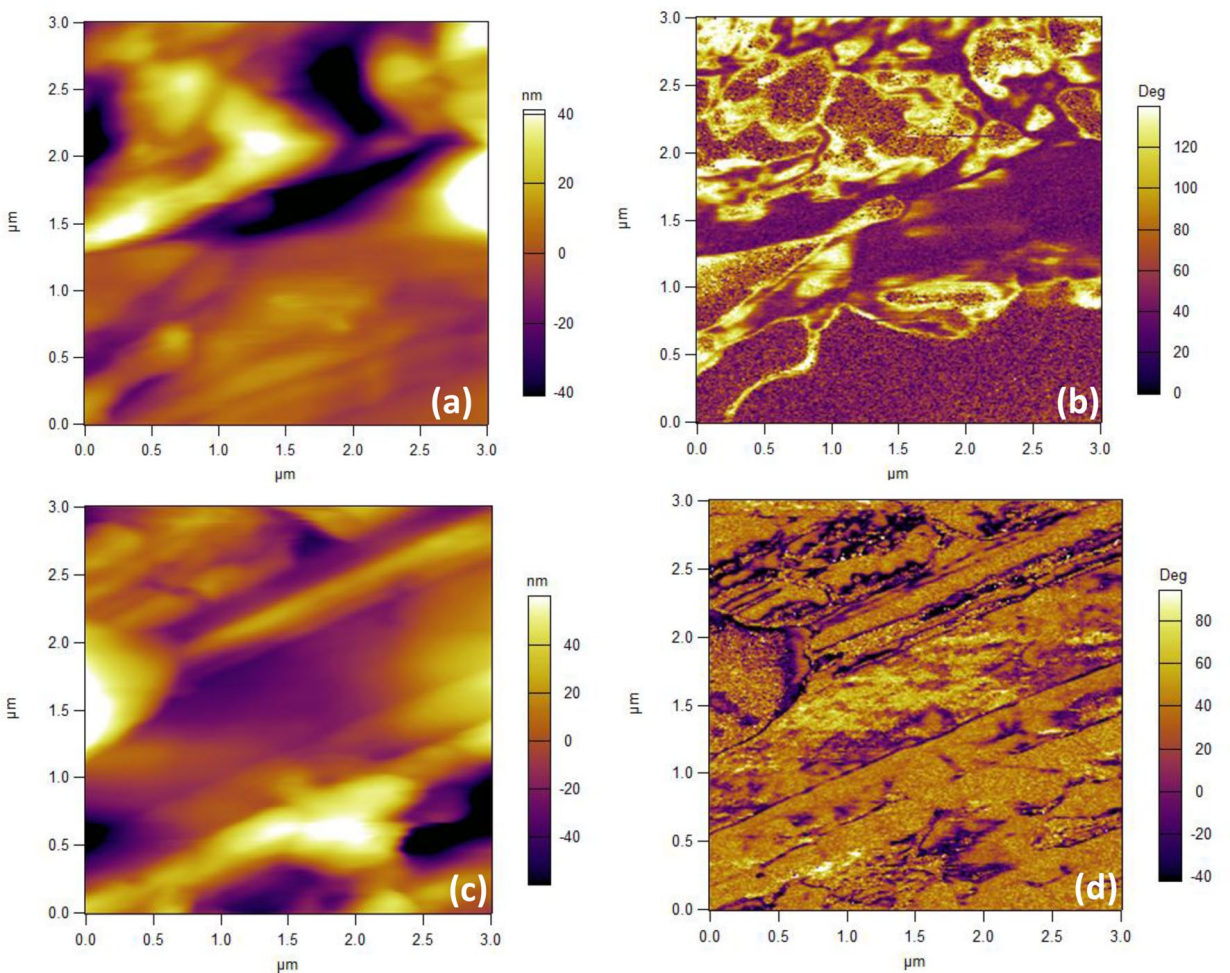
Topography and the corresponding phase image at (**a**, **b**) 278 K (**c**, **d**) 300 K.

It is understood that hysteretic phase switching alone does not always confirm ferroelectricity. Therefore, we have also performed temperature dependent ferroelectric domain imaging on the surface of ${\text {Sn}}_{0.7}{\text {Ge}}_{0.3}{\text {Te}}$. In Fig. 2 we present the topographic images and the corresponding PFM phase images. The phase image in Fig. 2b corresponds to the topographic image in Fig. 2a, both captured at 278 K. As it can be seen, the phase image displays features that are completely different from the topographic features. The dark–bright contrast in the phase image represents the local domains with differently oriented electric polarization vectors. At 300 K, the phase image (Fig. 2d) doesn’t show prominent ferroelectric domains and the contrast is due to topographic cross-talk alone (see Fig. 2c). Therefore, it is clear that the ferroelectric properties of the system are dramatically suppressed at elevated temperatures (room temperature in this case). This further confirms that ${\text {Sn}}_{0.7}{\text {Ge}}_{0.3}{\text {Te}}$ hosts local ferroelectricity with a Curie temperature comparable to the room temperature.

To investigate the origin of this temperature dependent ferroelectric behavior, we have performed Pair Distribution Function (PDF) analysis in the temperature range of 250–300 K. Finely ground power samples of ${\text {Sn}}_{0.7}{\text {Ge}}_{0.3}{\text {Te}}$ were used to perform PDF analysis. The powder was poured into a capillary with diameter of $\sim 0.6$ mm and the ends were sealed using adhesive. Perkin Elmer detector was used to obtain scattering data. Prior to every data set, dark measurement was performed. To obtain background data, scattering from empty capillary was recorded. Lanthanum Hexaboride (${\text {LaB}}_6$) standard was used for calibration of ${\text {Q}}_{damp}$ and other instrumental parameters. The beam spot size of $0.5 \times 0.5{\text { mm}}^2$ with a constant energy of 59.83 keV was taken. Processing data using fit2D and PdfgetX3^38^ provided G, which corresponds to probability of finding atoms at certain distances *r* in the material. G is obtained using Fourier transforming the scattering structure function F(Q)^39^, $G = \dfrac{2}{\pi } \int _{Q_{min}}^{\infty } F(Q)sin(Qr)dQ$ where Q stands for momentum transfer of the scattering particle. F(Q) is given as ${\text{ F }}({\text{ Q }})= {\text{ Q }}[{\text{ S }}({\text{ Q }})-1]$, with S(Q) being the structure function. Fitting of the PDF data is done using PDFgui^40^ software. All the datasets were initially simulated using a rock-salt cubic model. The refinement parameters were the scale, linear *r* dependence, lattice parameter, and the Atomic Displacement Parameter (${\text {U}}_{iso}$) values. The first peak of G vs *r* plot represents the nearest atom–atom correlations, similarly the second peak corresponds to second neighboring atom correlations (i.e., cation–cation or anion–anion distance) and so on. To investigate the local distortion in the structure, ${\text {U}}_{iso}$, lattice parameter obtained from cubic fit were fixed. The *r* range was taken 2.5–3.5 Å. The temperature evolution (250–300 K) of PDF over a range of 2.5–20 Å at 10 K intervals is shown in Fig. S3a. As the temperature increases, the thermal vibrations of atoms inside the lattice increases, which is observed in the decrease of peak intensities and increase in the peak width. The lattice parameter of ${\text {Sn}}_{0.7}{\text {Ge}}_{0.3}{\text {Te}}$ also increases from $\sim 6.165$ Å at 250 K to $\sim 6.171$ Å at 300 K (Fig. S3b). The atomic displacement parameter (${\text {U}}_{iso}$) ranges from 0.030 to 0.033 Å$^2$ for cations (Sn/Ge) whereas for Te it ranges from 0.019 to 0.022 Å$^2$ for 250–300 K region (Fig. S3c). The ${\text {U}}_{iso}$ provides a quantitative description of the vibrations of the atoms inside the lattice and its increase with temperature concords with the increase (decrease) in peak width (intensities) with temperature (Fig. S3a).

**Figure 3:**
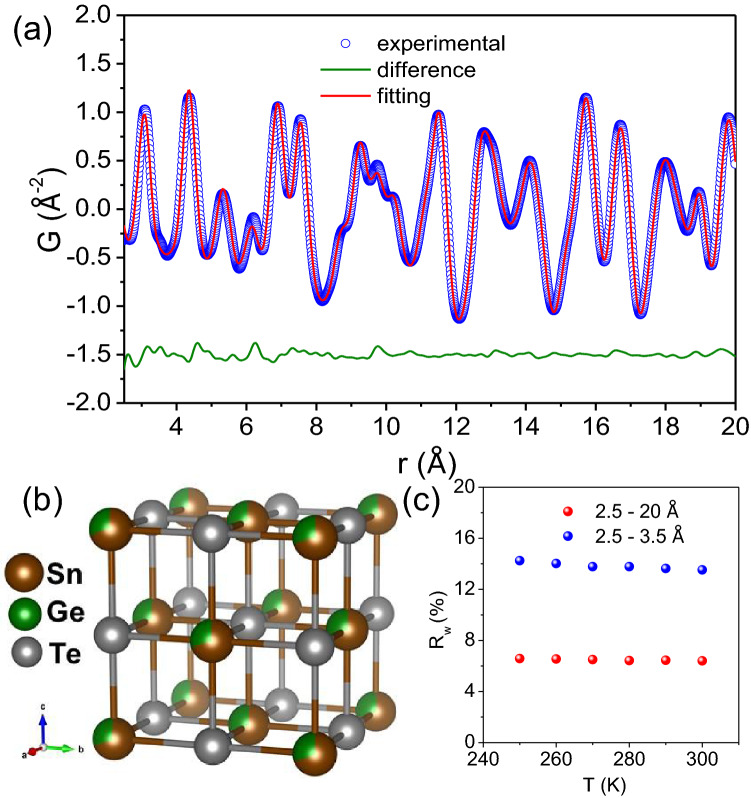
(**a**) X-ray pair distribution function (PDF) plot of ${\text {Sn}}_{0.7}{\text {Ge}}_{0.3}{\text {Te}}$ measured at 290 K and is fitted using cubic $Fm\bar{3}m$ model. (**b**) Cubic $Fm\bar{3}m$ structure of ${\text {Sn}}_{0.7} {\text {Ge}}_{0.3}{\text {Te}}$ (**c**) Temperature dependent Goodness of fit ($R_w$%) value for the total structure (2.5–20 $\AA$) and local structure (2.5–3.5 $\AA$) of ${\text {Sn}}_{0.7}{\text {Ge}}_{0.3}{\text {Te}}$ fitted with ${Fm}\bar{3}m$ model.

Being a total diffraction technique PDF analysis can parallelly provide information regarding any structural evolution on both global and atomic length scales^41^. PDF analysis revealed that ${\text {Sn}}_{0.7}{\text {Ge}}_{0.3}{\text {Te}}$ resides globally in rock-salt cubic lattice throughout the measured temperature range. When fitted with $Fm\bar{3}m$ model with partial occupancy of Sn/Ge in the cation site and Te in the anion site, the total PDF (2.5–20 Å) near the room temperature ( $\sim 290$ K) agrees reasonably well with the simulated curve (Fig. 3a). An excellent ${\text {R}}_w$ value of $\sim 6.6$% at 250 K obtained for the whole range fitting indicates that the global structure remains unperturbed with Ge alloying in SnTe (Fig. 3b). With increase in temperature, the ${\text {R}}_w$ value further decreases to $\sim 6.4$% at 300 K (Fig. 3c).

**Figure 4:**
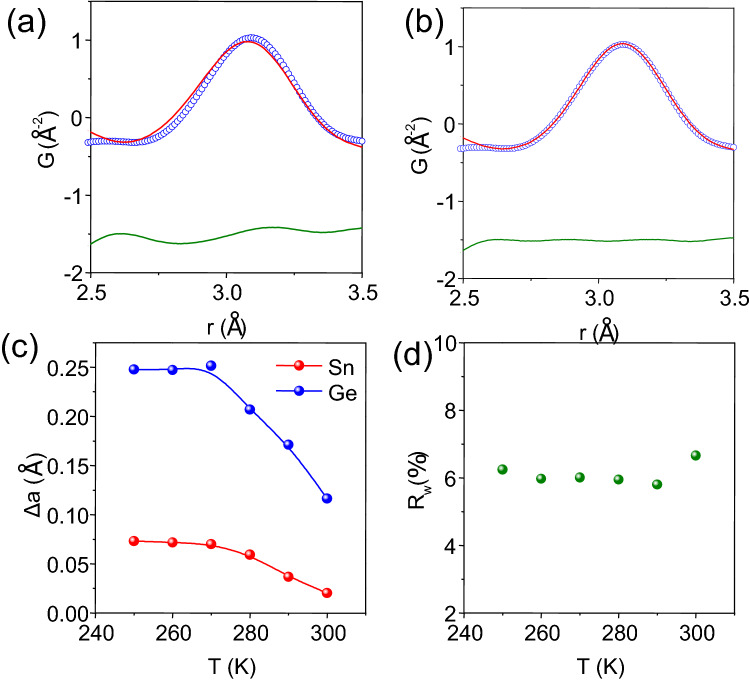
300 K PDF first peak fitted using (**a**) cubic $Fm\bar{3}m$ model and (**b**) Distorted model. (**c**) Magnitude of local distortion for Sn and Ge atoms. (**d**) Temperature dependent Goodness of fit $R_w$% value for local structure (2.5–3.5 Å) of ${\text {Sn}}_{0.7}{\text {Ge}}_{0.3}{\text {Te}}$ fitted with distorted model.

To unravel the nearest neighbor correlation in ${\text {Sn}}_{0.7}{\text {Ge}}_{0.3}{\text {Te}}$, that is the local bonding between cations (${\text {Sn}}^{2+}/{\text {Ge}}^{2+}$) and anion (${\text {Te}}^{2-}$), we have examined the bonding characteristics of the first peak (2.5 Å < r < 3.5 Å). When fitted using cubic model with parameters obtained from the total PDF fitting, the simulated fit does not correctly describe the local structural features (Fig. 4a). As shown in Fig. 3c, the ${\text {R}}_w$ value for the first peak (2.5–3.5 Å) of ${\text {Sn}}_{0.7}{\text {Ge}}_{0.3}{\text {Te}}$, using an undistorted cubic model is as high as $\sim$ 14.2% at 250 K and decreases gradually to 13.5% at 300 K. Such poor description of the first peak is most likely attributed to local distortion in ${\text {Sn}}_{0.7}{\text {Ge}}_{0.3}{\text {Te}}$. On off-centering Sn and Ge from their parent position, the fit for the nearest neighbor drastically improves (Fig. 4b). The ${\text {R}}_w$ for first peak fitting at 250 K drops to $\sim$ 6.2% (from 14.2%), when Sn and Ge are allowed to off-center from their mean position.

Interestingly, we observed that while Sn remains slightly off-centered ($\sim$ 0.07 Å) from its mean position at 250 K, Ge is found to be off-centered by a much greater extent ($\sim$ 0.24 Å) along the rhombohedral [111] direction in the global cubic SnTe. Such off-centering of Ge is a resultant of strong stereochemical activity of ${\text {4s}}^2$ lone pair on ${\text {Ge}}^{2+}$ which exerts a significant repulsion on the neighboring bond-pairs. Mention must be made that the lone pair of ${\text {Ge}}^{2+}$ in pure GeTe resides in a global rhombohedral structure (ferroelectric phase) up to 623 K^42,43^. Hence, in a symmetrical rock-salt type octahedral environment (in SnTe present case), the Ge will tend to off-center along the rhombohedral direction by expressing its ${\text {4s}}^{2}$ lone pair which we observe in present PDF experiments and predicted by previous DFT calculation^19^. Such locally off-centered Ge cations will induce charge polarization that can give rise to local ferroelectric instability as seen from PFM measurements.

Temperature evolution of the local structure shows dynamic off-centering of cations. Ge remains off-centered by $\sim$ 0.25 Å at 270 K and then starts to decrease the off-centering near the ferroelectric ${\text {T}}_c$ (Fig. 4c) in ${\text {Sn}}_{0.7}{\text {Ge}}_{0.3}{\text {Te}}$, whereas, Sn distorts only by $\sim$ 0.07 Å along rhombohedral direction at 250 K. At 300 K Ge and Sn shows local displacement of about 0.11 Å and 0.02 Å respectively. Low ${\text {R}}_w$ values for locally off-centered Sn/Ge (Fig. 4d) compared to undistorted Sn/Ge (Fig. 3c) indicates that the structure does reside as a low symmetric entity locally, although globally it averages out as a symmetrical rock-salt lattice. Such temperature dependent local distortion corroborates the PFM observations of local polarization and subsequent ferroelectric instability.

## Conclusion

In conclusion, we have demonstrated local ferroelectric polarization in ${\text {Sn}}_{0.7}{\text {Ge}}_{0.3}{\text {Te}}$ near room temperature by piezoresponse force microscopy and switching spectroscopy. We ascribe the presence of such local ferroelectric instability to the local off-centering of ${\text {Ge}}^{2+}$ along the rhombohedral direction ($<111>$) in the global rock-salt SnTe lattice, which is confirmed by synchrotron X-ray PDF investigations near room temperature. The off-centering of ${\text {Ge}}^{2+}$ is due to its stereochemically active $4{\text {s}}^2$ lone pair of electron which distorts the local octahedral coordination and forms local chain type off-centered region in global cubic lattice. We observe that below room temperature this local off-centering is significant and decreases near room temperature, thus corroborating with our PFM measurements. Local off-centering of cation induces ferroelectric instability, thereby softens the optical phonon modes which is prime reason to exhibit ultra-low lattice thermal conductivity in ${\text {Sn}}_{0.7}{\text {Ge}}_{0.3}{\text {Te}}$.

## Supplementary Information

Supplementary Information.
