# Supplementary material for: Local ferroelectric polarization switching driven by nanoscale distortions in thermoelectric Sn0.7Ge0.3Te
Source: Sci Rep. 2021 Aug 25;11:17190. doi: 10.1038/s41598-021-96299-3 (PMC8387443; doi:10.1038/s41598-021-96299-3)
Supplement: Supplementary file 1 — Supplementary Information. [file 41598_2021_96299_MOESM1_ESM.pdf]

Supplementary Material for the Local ferroelectric polarization switching driven by nanoscale distortions in thermoelectric $Sn_{0.7}Ge_{0.3}Te$

Aastha Vasdev[1*], Moinak Dutta[2,*] Shivam Mishra[1], Veerpal Kaur[1], Harleen Kaur[1], Kanishka Biswas[2], and Goutam Sheet[1†]

[1]*Department of Physical Sciences, Indian Institute of Science Education and Research Mohali, Sector 81, S. A. S. Nagar, Manauli, PO 140306, India and*

[2]*New Chemistry Unit, Jawaharlal Nehru Centre for Advanced Scientific Research, Bengaluru (Karnataka), India*

* Equal contribution first author

† Corresponding author: goutam@iisermohali.ac.in

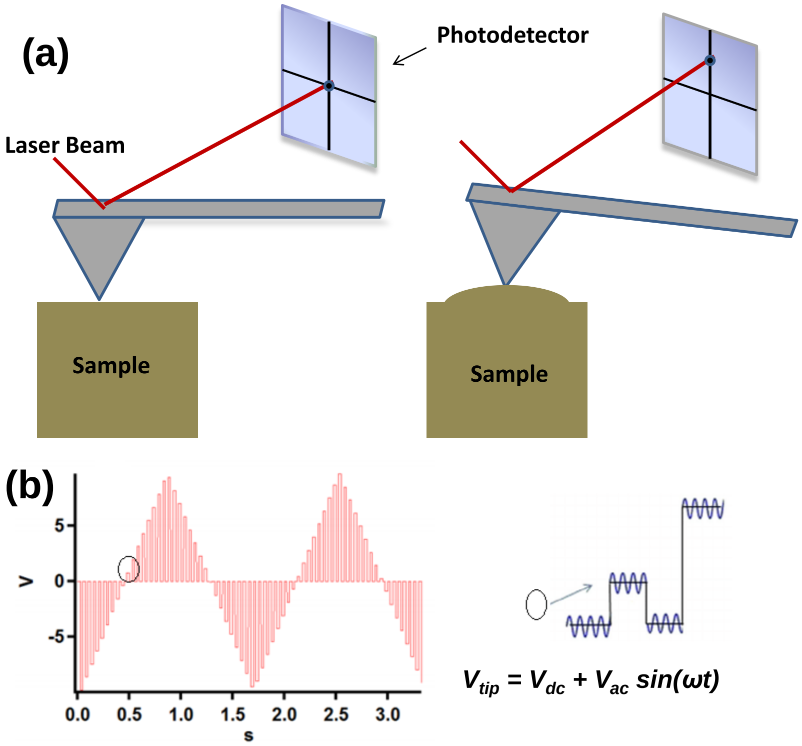

FIG. S1: (a) Schematic diagram of the Piezoresponse force microscopy (PFM) technique (b) Triangular pulsed signal used for biasing the tip in the DART-PFM spectroscopy mode

Methods:

Reagents: Tin (Alfa Aesar 99.99+%), germanium (Aldrich 99.999%) and tellurium (Alfa Aesar 99.999+%) were used for synthesis without further purification.

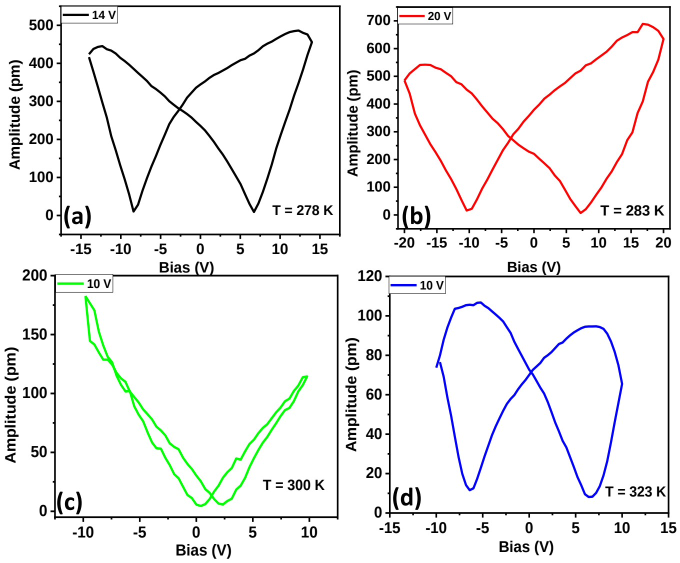

FIG. S2: Amplitude vs V_{dc} switching curve at temperature (a) 278 K (b) 283 K (c) 300 K and (d) 323 K

Synthesis: High quality polycrystalline ingots of $Sn_{1-x}Ge_xTe$ (x = 0 - 0.5) have been synthesized by melting the stoichiometric amount of Sn, Ge and Te in vacuum sealed (10^{-5} Torr) quartz tube. The tubes were kept vertically in

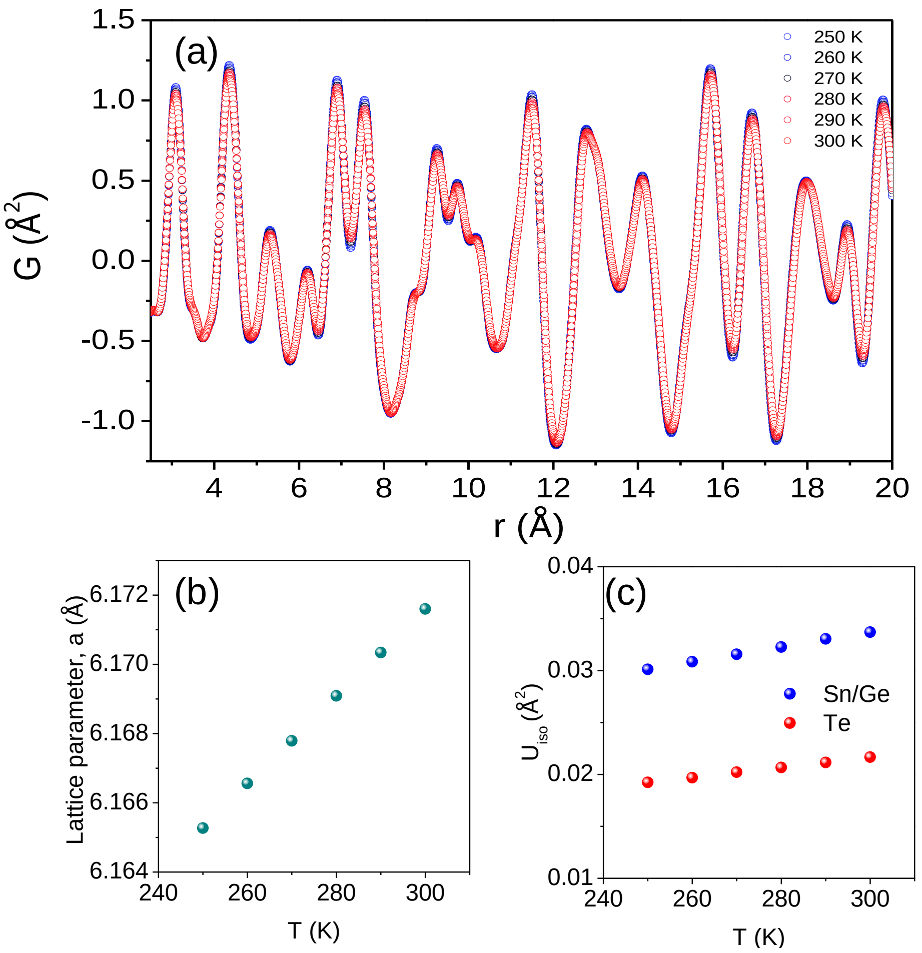

FIG. S3: (a) Temperature variation of X-ray PDF plot of $Sn_{0.7}Ge_{0.3}Te$ showing a decrease in intensity with increasing the temperature. (b) Temperature dependent lattice parameter. (c) Temperature dependent U_{iso} values.

a box furnace and slowly heated to 900°C over 12 hrs, then kept for 10 hrs, and cooled slowly (1.2^{o}C/min) to room temperature over a period of 12 hrs.

Figure S2 shows the temperature dependence of the "butterfly loop" in amplitude vs. field curve.

Synchrotron X-ray Pair Distribution Function (PDF): Finely ground powder samples of $Sn_{0.7}Ge_{0.3}Te$ were used to perform PDF analysis measured at PETRA III, P02.1, DESY, Germany. The powder was poured into a capillary with diameter of $\sim$ 0.6 mm and the ends were sealed using adhesives. Perkin Elmer detector was used to obtain scattering data. Prior to every data set, dark measurement was performed. To obtain background data, scattering from empty capillary was recorded. Lanthanum Hexaboride (LaB_6) standard was used for calibration of Q_{damp} and other instrumental parameters. The beam spot size of 0.5 x 0.5 mm^2 with a constant energy of 59.83 keV was taken.

Processing data using fit2D and PdfgetX3[1] provided G, which corresponds to probability of finding atoms at certain distances r in the material. G is obtained using Fourier transforming the scattering structure function F(Q)[2]

$$G = \frac{2}{\pi}\int_{Q_{min}}^{\infty} F(Q)Sin(Qr)dQ,$$

Here Q stands for momentum transfer of the scattering particle. F(Q) is given as F(Q) = Q[S(Q)-1], with S(Q) being the structure function.

Fitting of the PDF data is done using PDFgui[3]software. All the datasets were initially simulated using a rock-salt cubic model. The refinement parameters were the scale, linear r dependence, lattice parameter, and the Atomic Displacement Parameter (ADP) values. The first peak of G vs r plot represents the nearest atom - atom correlations, similarly the second peak corresponds to second neighboring atom correlations (i.e., cation - cation or anion - anion

distance) and so on. To investigate the local distortion of the cations, ADPs, lattice parameter obtained from cubic fit were fixed. The r range was taken 2.5 Å to 3.5 Å.

---

[1] P. Juhas, T. Davis, C.L. Farrow, S. J. L. Billinge, PDFgetX3 *Journal of Applied Crystallography* ,**46(2)**, 560-566(2013).
[2] T. Proffen, S. J. L. Billinge, T. Egami, D. Louca, *a practical guide. In Zeitschrift für Kristallographie - Crystalline Materials* , **218**, p 132(2003).
[3] C. L. Farrow, P. Juhas, J. W. Liu, D. Bryndin, E. S. Božin, J. Bloch, P. Th, S. J. L. Billinge, , PDFfit2 and PDFgui *Journal of Physics: Condensed Matter* , **19(33)**, 335219(2007).
